# Valproic acid induced necrohemorragic pancreatitis: Case report and diagnostic approach in uncommon pancreatitis

**DOI:** 10.1016/j.ijscr.2019.05.052

**Published:** 2019-06-04

**Authors:** Silvia Carolina Barbosa, Paulo Cabrera, Bayron Guerra, Carlos F. Roman

**Affiliations:** Fundacion Cardioinfantil, General Surgery Department, Bogota, Colombia

**Keywords:** Acute pancreatitis, Valproic acid, Necrotizing pancreatitis, Medication induced pancreatitis, Algorithm

## Abstract

**Background:**

Acute pancreatitis (AP) is one of the most frequent gastrointestinal alterations in the United States. Medication induced AP has been undervalued in the surgical environment as etiologic entity of necrohemorragic pancreatitis (NHP). A case of NHP induced by valproic acid (VA) is presented, and an exclusion diagnostic algorithm is proposed.

**Case report:**

A 29-year-old female with past medical history of migraine controlled with VA was admitted to our institute for acute abdominal pain and peritoneal irritation. An exploratory laparotomy was performed, finding pancreatic necrosis and a diagnosis of NHP was determined. The most frequent etiologies for AP were discarded. An exclusion diagnostic algorithm was performed reaching VA as etiologic gent. The treatment consisted on medication withdrawal, oral restriction, parenteral nutrition, hydration, analgesia and peritoneal wash-outs with a positive outcome.

**Conclusion:**

Is convenient to consider a medication induced AP in patients without clear causative agent, such as the VA case presented. An etiological diagnosis algorithm of exclusion is proposed, for an adequate therapeutic approach in medication induced PNH. Algorithm validation is required.

## Highlights

• Is convenient to consider a medication induced necrohemorragic pancreatitis in patients without clear causative agent.
• Because progressive increase in AP incidence and the impact on morbidity and mortality is crucial determine an etiologic diagnosis in order to proceed to appropriate therapeutic interventions as for recurrence prevention.
• The valproic acid is a strong AP inducer (class Ia) in its evidence based classification and review of literature on medication induced AP, with the biomolecular evidence of pancreatic injury.
• An adequate initial approach with a complete clinical history and pharmacological background, good physical examination, and pertinent extension of laboratory tests are necessary in order to achieve a proper AP etiology.
• The etiologic diagnosis algorithm for AP proposed could be considered as a diagnostic exclusion tool, and is easy to apply for a timely therapeutic approach in medication induced AP.

## 1 Introduction

Acute pancreatitis (AP) is one of the main causes of hospital admissions for gastrointestinal disorders in the US with approximately 230,000 admissions per year [1,2]. Valproic acid (VA) induced AP has been previously documented by multiple case reports and epidemiological studies [3]. This medication has been widely implemented for seizure disorder control, especially in children. In Colombia the VA is the most frequently used medication for seizure control with up to 33% of all treatments [4]. Moreover, VA is widely used for migraine prophylaxis in adults [5] more frequently used in women [6].

A necrohemorragic pancreatitis (NHP) case is described on a 29-year-old patient, without a clear etiology, considering VA intake as causative agent. Given the infrequency of the medication induced AP an exclusion diagnostic etiologic algorithm is proposed for AP. This case report has been structured according to the international SCARE guidelines [7]. A descriptive literature review was performed for the algorithm design, and patients clinical history, physical examination, the biomolecular mechanisms and the epidemiologic factors were considered.

## 2 Case report

A 29-year-old patient was admitted to our institute, due to 6 h of sporadic acute abdominal pain located on epigastrium and mesogastrium associated to nausea and vomiting. Upon admission the patient denies history of trauma or clear pain triggering factors. The patient had a past medical history of migraine syndrome controlled with VA for the last 2 years (dose of 250 mg/12 h), cavernous hemangioma on the left side of her face with multiple reconstructive surgeries and a family history of rheumatoid arthritis in her mother. The patient denied smoking or alcohol intake.

On admission the patient had normal vital signs, and her physical examination revealed peritoneal irritation signs, absent bowel sounds, no skin lesions, and no mases were felt on abdominal palpation.

Positive laboratory work-up results revealed leukocytosis (13.000), a metabolic acidosis on arterial gases (pH: 7.34, FiO_2_: 21%, PCO_2_: 31.8, PO_2_: 65.9, HCO_3_: 17.9, BE: −9.3), hyperlactatemia (2.7) and amylase at 270 U/L (30–118 U/L). No alterations were detected on abdominal ultrasound.

**Fig. 1:**
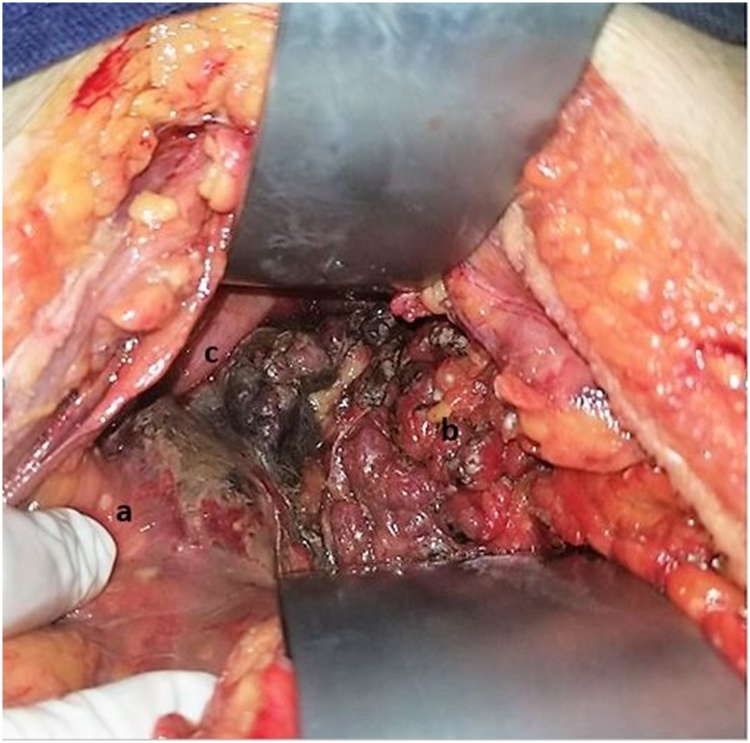
Intraoperative findings of Valproic Acid induced Acute Pancreatitis (AP). Evidence of a: steatonecrosis foci on parietal peritoneum; b: peripancreatic fat; c: pancreatic necrosis at the head of the pancreas.

The patient was taken to an exploratory laparotomy due to a clinical acute surgical abdomen. Intraoperative findings included necrohemorragic intra- and retroperitoneal fluid, extending to omental bursa and periduodenal space, with segmental epiploic necrosis, multiple stheatonecrotic foci on the pancreatic cell, and superficial head, body, and tail pancreatic necrosis (Fig. 1), requiring negative pressure system (VAC Abthera^®^) placement in pancreatic cell. No stones were felt on gallbladder palpation.

During hospital stay bile stones and alcoholism were discarded upon AP etiologic approach. Further extension work-up excluded other etiologies for AP such as infection, trauma, hypercalcemia, tumors, hypertriglyceridemia, or autoimmunity. Normal results were obtained on triglycerides, electrolytes, antinuclear antibodies, complement proteins (C3, C4), hepatitis B virus anticore IgM, hepatitis B superficial antigen, HIV antibodies, and abdominal CT scan.

The use of VA was considered as the etiologic factor for the patients’ AP, due to no evidence of other abnormalities on clinical work-up.

Immediate VA withdrawal was ordered, and oral intake restriction, with parenteral nutrition to meet daily nutritional requirements, and pain management was performed. Two additional surgical procedures were required: Negative pressure system change with peritoneal washout, and a posterior formal abdominal closure requiring a Jackson-Pratt drain placed at pancreatic cell.

The in hospital control workout did not report abnormal values, and CRP levels progressively decreased as inflammatory modulation marker.

A postoperative cholangioresonance reported a normal density pancreatic tissue, a 13 mm image suggesting a non-significant hematoma, and a pancreatic fistula was discarded through drainage amylase level. Other non-significant small collections were identified: sub hepatic collection of 31 × 16 mm, another perigastric collection of 20 × 15 mm, and a periumbilical collection of 48 × 24 mm.

During hospital stay the patient went through a poor pain modulation due to a psychoafective disorder, increasing the hospital stay. The patient underwent through a slow but positive recovery, and had a hospital length of stay (LOS) of 18 days. The patient was discharged with recommendations and pain medication. An abdominal CT was performed as follow-up image, and revealed complete collection resolution, without other abnormal findings were seen.

A narrative literature review on infrequent AP etiologies in adults (once bile stones and alcoholism were excluded) identified different association on medication induced AP on multiple systemic reviews. Table 1 reports the Badalow classification [8] on medications with clear effects on AP, according to their epidemiologic incidence on literature.

**Table 1:** Medications associated with acute pancreatitis according to Badalov et al. classification.

Definition	Example
**Class I drug Ia:** at least one case report evidence of a positive rechallenge, and exclusion of other causes of AP	Codeine, cytarabine, dapsone, enalapril, furosemide, isonaizid, mesalamine, metronidazole, pentamidine, pravastatin, procainamide, simastatin, Sulfamethoxazole, sulindac, tetracicline, **valproic acid**.
**Ib:** Similar to class Ia except that other causes of AP could not be ruled out	Amiodarone, azathioprine, dexamethasone, ifosfaide, lamivudine, losartan, 6-MP, premarine, TMP-SMZ.
**Class II drugs** Include at least four case reports with a consistent latency period for at least 75% of the cases	Acetaminophen, clozapine, erythromycin, estrogen, 1-asparaginase, propofol, tamoxifen.
**Class III drug** at least two case reports but do not have re-challenge data or a consistent latency period	Alendronate, carbamazepine, ceftriaxone, clarithromicyne, cyclosporin, hidrochlorothiazide, interferone, ribavirin, metformin, minocycline, naproxen, paclitaxel, prednisone, prednisolone.
**Class IV drug** One case report without re-challenge data	Ampicillin, cisplatin, colchicine, cyclophosphamide, diclofenac, doxorubicin, Interleukin2, octreotide, propoxyphene, rifampin, risperidone, sertaline, Tacrolimus, vincristine.

AP(acute pancreatitis); 6MP(mercaptopurine); TMP-SMX(trimethoprim sulfamethoxazole).

Taken from: Contemporary review of drug-induced pancreatitis: A different perspective World J Gastrointest Pathophysiol 2014 November 15.

**Fig. 2:**
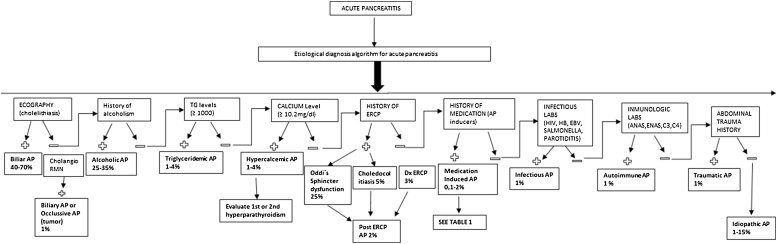
Etiological diagnosis algorithm of acute pancreatitis. AP: acute pancreatitis; CHOLANGIORMN; cholangioresonance; TG: triglycerides; ERCP: endoscopic retrograde cholangiopancreatography; Dx: diagnostic; LABS: laboratory tests; HIV: human immunodeficiency virus; HBV: hepatitis B virus; EBV: Epstein Barr virus; ANAS; antinuclear antibodies; ENAS: extractable nuclear antibodies; C3-C4: complement proteins.

The infrequent AP etiology, when common causes are discarded, is a challenging exclusion diagnostic process. This lead our research group to design an etiologic diagnostic algorithm, according to the epidemiological frequencies in the light of actual evidence (Fig. 2).

## 3 Discussion

There has been a progressive increase in AP incidence, and its impact on morbidity and mortality is determined by a proper therapeutic approach [9]. It is crucial to determine an etiologic diagnosis in order to proceed to appropriate therapeutic interventions as for recurrence prevention. A medication induced AP has to be considered as etiologic agent upon a patient without clear etiology, defined by absence of bile stones and alcoholism, with normal triglycerides levels, and absence of tumors or anatomic variations [10].

Is widely known that the medication induced AP is an exclusion diagnosis, as the case presented. However, it should be noted that its initial clinical presentation may not meet the diagnostic criteria proposed by the Atlanta classification 2012 for AP [11]. The presented case had no typical abdominal band pain, the amylase level was not greater than 3-fold for the laboratory parameter (270 U/L with a higher laboratory value of 118 U/L), and no imaging changes were seen on admission (ultrasound without alteration). All the above, and considering a young patient with acute abdominal pain, leukocytosis, metabolic acidosis, and absent bowel sounds, a surgical exploration was performed, evidencing the aforementioned findings compatible with PNH. This procedure increases hospital LOS as well as the risk of morbidity and mortality when compared to patients undergoing uncomplicated AP [2].

Biliary stones and alcohol are responsible for more than 90% of all cases of AP in adults [9]. However, once these 2 factors are ruled out as the etiologic agents in AP, multiple additional studies are required for precise diagnosis and prompt therapeutic interventions. Within that 10% of missing etiological diagnoses, medications are responsible for 0.1–2% [1,3].

There were no clear causes for AP on further work-up in the presented case. The presence of biliary stones was ruled out on clinical history, ultrasound, and cholangioresonance. Alcohol abuse or tobacco use was ruled out as other toxins that may be directly related to AP [2]. There was no evidence of mechanical factors such as pancreas divisum (PD) whose prevalence is similar in those patients with and without idiopathic (7.5%) and alcoholic (7%) pancreatitis, determining that PD by its self does not causes the disease [2]. A complete metabolic, infectious and immunological profiles ruled out hypertriglyceridemia, infectious processes, hypercalcemia, pregnancy, vascular or autoimmune diseases. Therefore, a medication induced AP was considered, secondary to chronic consumption of VA.

VA has been considered an infrequent etiology of AP with a general incidence of 1:40.000 cases [12]. Although, pediatric population are more commonly affected by VA induced AP [12], there is evidence of multiple cases reported since 1979 by Batalden et al. with some fatal cases [9,12,13]. Therefore, the real VA induced AP incidence might be underestimated due to lack of registration or investigation.

In addition, Badalov et al. [8] in New York (2007), reported VA as a strong AP inducer (class Ia) in its evidence based classification and review of literature on medication induced AP. This means that the VA (class Ia) presents at least one case report of recurrent AP at re-challenge with this drug, having excluded other AP causes [3,8]. This classification provides an important and rapid reference of medication induced AP (Table 1).

The World Health Organization through the Uppsala Monitoring Center conducted a hospital analysis in patients with medication induced AP between 2006 and 2016. This analysis reported VA as the most frequent medication inducing AP, representing 4.7% of the total number of patients with this condition [14]. This suggests that AP induced by VA represents a higher incidence than previously reported [14]. Additionally, other studies report greater sensitivity of serum lipase than of serum amylase for VA induced AP [10]. This may explain the amylase level on our case (less than 3 times the higher value), and lipase is not routinely performed at admission. The absence of elevation of amylase level above 3 times the superior value does not exclude the medication induced AP. Therefore, its crucial to highlight that there is no specific biochemical alteration on VA induced AP [10]. The medical personnel must consider this concept in the context of a patient with severe abdominal pain and history of VA consumption, for an optimal diagnostic approach, and avoid unnecessary procedures.

Given the above, multiple biomolecular studies are being developed on the effect of VA on pancreatic tissue. The pathophysiological mechanisms of pancreatic injury of VA in AP were poorly understood in 2013. The direct toxic effect of this medication was attributed to free oxygen radicals in pancreatic tissue, associated to a deficit of superoxide dismutase (SOD), catalase and glutathione peroxidase (GPO) (responsible for removing free radicals) [1]. Currently, it has been reported that histone deacetylases (HDAC) is responsible for mediating the development of the pancreas and contribute to its recovery after an injury. VA predisposes to pancreatitis by inhibiting HDAC and causing an imbalance in pancreatic recovery [15]. In an experimental model of pancreatic injury, VA delayed recovery of the pancreas and reduced the proliferation of acinar cells by inhibiting HDAC activity. This leads to the over-expression of gene Sox9 involved in pancreatic and duodenal development, acting in the endocrine development of ductal and centroacinate cells and the sustained b-catenin nuclear activation that delays pancreatic regeneration [15,16].

All of the above supports that there is sufficient evidence to consider VA as a potent AP inducer, given its strong association, high frequency, and biomolecular evidence of pancreatic injury.

On the other hand, within the hospital context, attention should be paid on the fact that no relationship could be established between the patient’s clinical condition and AP diagnosis, as presented in this case. This rises the need for an etiological diagnosis definition of AP without apparent cause. Therefore, a diagnostic algorithm is presented in an attempt for an etiology approach in order to reach timely therapeutic maneuvers in AP, and impact morbidity. It is clear that this algorithm requires clinical validation prior to its implementation. However, it could be considered as a diagnostic tool for patients with AP without clear etiology. This algorithm allows an etiologic diagnosis approach for AP through simple clinical and laboratory variables, locating the possible diagnosis by epidemiological frequencies according to the available evidence [9]. To our knowledge, this is the first etiological diagnosis algorithm in the literature proposed for AP without clear cause.

This case report draws attention to all medical personnel facing AP without a clear cause, and a possible medication etiology should be considered; being the VA one of the most frequent and stronger AP inducers. An adequate initial approach with a complete clinical history and pharmacological background, good physical examination, and pertinent extension of laboratory tests are necessary in order to achieve a proper AP etiology.

## 4 Conclusion

A medication etiology should be timely suspected in patients going through AP without a clear etiology. VA emerges as a determinant medication in the induction of AP from the epidemiological and biomolecular point of view. The etiologic diagnosis algorithm for AP proposed could be considered as a diagnostic exclusion tool, and is easy to apply for a timely therapeutic approach in medication induced AP. Validation of this algorithm is required.

## Conflicts of interest

The authors have no financial or proprietary interest in the subject matter, we do not have any conflict of interest.

## Source of funding

There was no financing aid or founding of any entity for this manuscript.

## Ethical approval

This article is exempt from ethical approval. Prior informed consent from the patient was obtained for this case report.

## Consent

Written informed consent was obtained from the Patient for the publication of this case report and accompanying images which is attached.

## Author’s contribution

Author 1: Silvia Carolina Barbosa Valenzuela, MD; Patient’s clinical history review, informed consent signature, literature review. Article build up an writing, with algorithm creation, conceptualization, investigation and visualization.

Author 2: Paulo Cabrera, MD; Article and algorithm review and build up. Patient's surgical procedure and clinical follow-up during his hospital stay and ambulatory monitoring, writing, Review, editing and supervision.

Author 3: Byron Guerra, MD; Article and algorithm review and build up. Patient's surgical procedure and clinical follow-up during his hospital stay and ambulatory monitoring, writing, Review, editing and supervision.

Author 4: Carlos Roman Ortega, MD; Article and algorithm review and build up. Patient's surgical procedure and clinical follow-up during his hospital stay and ambulatory monitoring writing, Review, editing and supervision.

## Registration of research studies

N/A.

## Guarantor

Author 1: Silvia Carolina Barbosa Valenzuela MD.

Author 2: Paulo Cabrera MD.

Author 3: Byron Guerra MD.

Author 4: Carlos Roman Ortega MD.

## Provenance and peer review

Not commissioned, externally peer-reviewed.
